# Coherent spin control of s-, p-, d- and f-electrons in a silicon quantum dot

**DOI:** 10.1038/s41467-019-14053-w

**Published:** 2020-02-11

**Authors:** R. C. C. Leon, C. H. Yang, J. C. C. Hwang, J. Camirand Lemyre, T. Tanttu, W. Huang, K. W. Chan, K. Y. Tan, F. E. Hudson, K. M. Itoh, A. Morello, A. Laucht, M. Pioro-Ladrière, A. Saraiva, A. S. Dzurak

**Affiliations:** 1 Centre for Quantum Computation and Communication Technology, School of Electrical Engineering and Telecommunications, The University of New South Wales, Sydney, NSW 2052 Australia; 2 Institut Quantique et Département de Physique, Université de Sherbrooke, Sherbrooke, Québec J1K 2R1 Canada; 3 QCD Labs COMP Centre of Excellence, Department of Applied Physics, Aalto University, 00076 Aalto, Finland; 4 School of Fundamental Science and Technology, Keio University, 3-14-1 Hiyoshi, Kohokuku, Yokohama 223-8522 Japan; 5 Quantum Information Science Program, Canadian Institute for Advanced Research, Toronto, ON M5G 1Z8 Canada; 6 Present Address: Research and Prototype Foundry, The University of Sydney, Sydney, NSW 2006 Australia

**Keywords:** Quantum dots, Qubits

## Abstract

Once the periodic properties of elements were unveiled, chemical behaviour could be understood in terms of the valence of atoms. Ideally, this rationale would extend to quantum dots, and quantum computation could be performed by merely controlling the outer-shell electrons of dot-based qubits. Imperfections in semiconductor materials disrupt this analogy, so real devices seldom display a systematic many-electron arrangement. We demonstrate here an electrostatically confined quantum dot that reveals a well defined shell structure. We observe four shells (31 electrons) with multiplicities given by spin and valley degrees of freedom. Various fillings containing a single valence electron—namely 1, 5, 13 and 25 electrons—are found to be potential qubits. An integrated micromagnet allows us to perform electrically-driven spin resonance (EDSR), leading to faster Rabi rotations and higher fidelity single qubit gates at higher shell states. We investigate the impact of orbital excitations on single qubits as a function of the dot deformation and exploit it for faster qubit control.

## Abstract (web summary)

Quantum dots are often referred to as “artificial atoms” as they create zero-dimensional traps for electrons, with characteristic atom-like spectra. Leon et al. demonstrate that higher shell and orbital states of a multi-electron silicon quantum dot with better control fidelity than single electron dots.

## Introduction

Qubit architectures based on electron spins in gate-defined silicon quantum dots benefit from a high level of controllability, where single and multi-qubit coherent operations are realised solely with electrical and magnetic manipulation. Furthermore, their direct compatibility with silicon microelectronics fabrication offers unique scale-up opportunities^1^. However, fabrication reproducibility and disorder pose challenges for single-electron quantum dots. Even when the single-electron regime is achievable, the last electron often is confined in a very small region, limiting the effectiveness of electrical control and interdot tunnel coupling. Many-electron quantum dots were proposed as a qubit platform decades ago^2^, with the potential of resilience to charge noise^3,4^ and a higher tunable tunnel coupling strength to other qubits^5^. In the multielectron regime, the operation of a quantum dot qubit is more sensitive to its shape. If it is axially symmetric, the orbital energy levels will be quasi-degenerate^6–8^, which is detrimental for quantum computing. On the contrary, if the quantum dot is very elongated, a regular shell structure will not form, and it becomes difficult to identify a priori what charge configurations will lead to spin-1/2 systems suitable for quantum computing^2,9,10^.

## Results

### Filling s-, p-, d- and f-orbitals in a silicon quantum dot

**Fig. 1:**
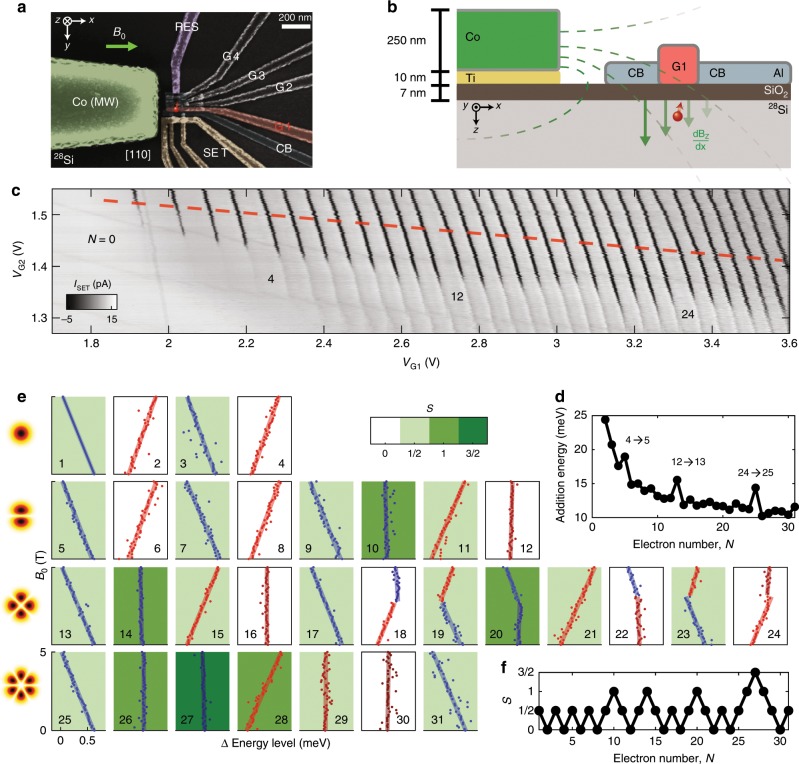
Device overview and electron occupancy measurement. **a** False-coloured SEM image of a nominally identical device to that reported here. A quantum dot is formed under gate G1 (red), in the location marked by the red symbol. Gate RES is connected to an n-doped reservoir to load/unload electrons to/from the quantum dot, with tunnel rates controlled by G2, G3 and G4. Gate CB serves as a confinement barrier. The cobalt (Co) structure at the left of the image acts as both a micromagnet and electrode for EDSR control (green). **b** Cross-sectional schematic of the device, fabricated on a purified Silicon-28 epi-layer (800 ppm). **c** Charge stability map of the quantum dot at *B*_0_ = 0 T, produced by plotting the pulsed lock-in signal from SET sensor *I*_pulse_ vs *V*_G1_ and *V*_G2_. A square wave with peak-to-peak amplitude of 2 mV is applied to G1 for lock-in excitation. Dynamic compensation is applied to the SET sensor to maintain a high read-out sensitivity. Electron numbers *N* for full shells are marked on the diagram. **d** Charging energies along the red line in (**c**) in the tightly confined regime. **e** Magnetospectroscopy of the first 31 electrons occupied in the quantum dot, up to *B*_0_ = 5 T, with background colour of each plot representing spin state *S* at *B*_0_ = 0 T. Change in addition energies with magnetic field are measured and fitted with straight lines. Since the charging energy is measured only from the second electron, the first electron is depicted by a straight line with no data. Each row of the array of plots belongs to the same shell, while each column has the same number of valence electrons in its outershell. The cartoon on the left gives an example of the electron wavefunction for each shell. **f** Spin state of each electron occupancy extracted from (**e**).

The scanning electron microscope (SEM) image in Fig. 1a shows a silicon metal-oxide-semiconductor (Si-MOS) device that forms a quantum dot at the Si/SiO_2_ interface under gate G1, separated from the reservoir by a barrier that is controlled by gate G2—see Fig. 1b for a cross-sectional representation. We first study the electronic structure of the dot from its charge stability diagram, using the technique from ref. ^11^, which maps out each electron transition between quantum dot and reservoir as a function of gate potentials. Figure 1c shows a well ordered set of electron transitions, revealing a quantum dot that can be occupied by up to 31 electrons with no significant evidence of disorder in the form of reconfiguration of charge traps in the oxide or at the Si/SiO_2_ interface (which would lead to the occurrence of addition energies that do not follow a periodic rule). This occupancy range is slightly better than other devices based on similar technology^12^. Additional charge transitions in Fig. 1c (faint nearly-horizontal lines) arise from states between the reservoir and the quantum dot and do not affect the qubit operation. Lowering the voltage of gate G2 confines the quantum dot further and changes its eccentricity in the *x*–*y* plane.

Following the red dashed line in Fig. 1c allows us to investigate the addition energies, i.e., the energy necessary to add the *N*-th electron to a dot that contains *N*−1 electrons, as plotted in Fig. 1d. The first noticeable effect is that the charging energy is roughly inversely proportional to the number of electrons, which is a consequence of the dot size becoming larger as the dot fills up. Furthermore, very distinct peaks appear at transitions 4 → 5, 12 → 13 and 24 → 25. To understand the significance of these electron numbers, one may refer to the Fock-Darwin energy levels^13,14^, where the internal spin (*↑*, *↓*) and valley (*v*_+_, *v*_−_) quantum numbers give the multiplicity of each orbital state in a two-dimensional quantum dot. As a result, a full shell is formed when there are 4, 12 and 24 electrons in the 2D quantum dot, and so an extra energy, corresponding to the orbital level splitting, must be supplied in order to begin filling the next shell. The filling of three complete electron shells has previously been observed in a GaAs quantum dot^6^, where the single-valley nature of the semiconductor leads to a filled third shell at *N* = 12 electrons, but until now has not been observed in a silicon device. The observed shell filling is analogous to the aufbau principle of atomic physics, that allows us to construct the electronic structure of many-electron atoms in terms of occupation of the atomic electron levels from bottom up.

As well as the large jumps in addition energy observed after complete shells are filled, a finer structure at intermediate fillings is also present due to the valley splitting *Δ*_VS_^15^, the energy difference between excitations along the major and minor axes of the elliptical quantum dot^16^ *Δ*_*xy*_, and electronic quantum correlations^17^, dominated by the exchange coupling *J*. These energy scales are much smaller than the shell excitation, so that we can identify each set of levels by a principal quantum number. Each shell is spanned by the valley^18–20^, spin and azimuthal^21^ quantum numbers. For this particular quantum dot, *Δ*_VS_ and *Δ*_*xy*_ may be estimated^12,19^ and both are of the order of hundreds of μeV, which is consistent with typical observations for quantum dots with similar designs^20^. Since both splittings are similar in magnitude, it is difficult to label the inner shell structure based solely on the addition energy diagram.

Magnetospectroscopy of the electron transitions (Fig. 1e) indicates the variation in total spin *S* between consecutive fillings by tracking the addition energy as a function of external magnetic field strength *B*_0_ (see ref. ^22^ for a detailed method of extracting total spin *S* from magnetospectroscopy). A negative slope indicates an increase in total *S*, and a positive slope represents a decrease in total spin. At lower electron occupancies, *S* alternates between $\frac{1}{2}$ and 0, with cumulative spin state *S* presented in Fig. 1f. This indicates that the sequential electron loading favours anti-parallel spin states, implying *J* ≪ *Δ*_*xy*_, *Δ*_VS_. As the electron number increases, Hund’s rule applies as some of the electrons are loaded as parallel spins (*S* = 1 or $\frac{3}{2}$ states), indicating *J* > *Δ*_*xy*_, *Δ*_VS_ in these cases. Notice that in a few instances, a kink is observed, which indicates that the Zeeman splitting has become larger than some orbital or valley splitting, so that the electron occupies the higher orbital and creates a state with higher *S*.

The observation of *S* = 1 spin states is potentially significant, in the context of the study of symmetry-protected topological phases of *S* = 1 spin chains with antiferromagnetic Heisenberg coupling. As conjectured by Haldane^23^, such *S* = 1 spin chains possess a fourfold degenerate ground state, protected by a topological gap to higher excited states. Finite-length chains exhibit fractionalized *S* = 1∕2 states at their ends, which could be exploited for robust quantum computing schemes^24–26^. The experimental realization of controllable *S* = 1 Haldane chains, however, has remained a formidable challenge^27^. In semiconductor quantum dots, methods to locally control and read-out chains of spins are now mature. Engineering *S* = 1 with the natural Heisenberg exchange interaction in this system might open exciting opportunities for future studies in this field.

### Operation of single-valence multielectron spin qubits

We now examine the spins of monovalent dot ocupations as potential qubits, i.e., the first electron of each shell *N* = 1, 5, 13 and 25, which we call s-, p-, d- and f-electrons, respectively, in reference to the electronic orbitals^28^. To demonstrate single-qubit control, we designed this device with the capability to perform electrically-driven spin resonance (EDSR). A cobalt micromagnet positioned near the quantum dot induces a magnetic field gradient. An external uniform magnetic field *B*_0_ = 1.4 T provides a Zeeman splitting between spin states for spin to charge conversion read-out^29^. This field also fully magnetises the micromagnet (cobalt is fully magnetised at *B*_0_ ~ 0.4 − 0.5 T), leading to a field gradient of ~1 T/μm in the direction transverse to the quantization axis. This provides the means to drive spin flips without the need for an AC magnetic field^30–32^. Instead, a  ~40 GHz sinusoidal voltage is applied directly to the magnet. The antenna-like structure creates an AC electric field at the quantum dot, so that the electron wavefunction oscillates spatially within the slanted magnetic field, which drives Rabi oscillations of the qubit^33–35^.

**Fig. 2:**
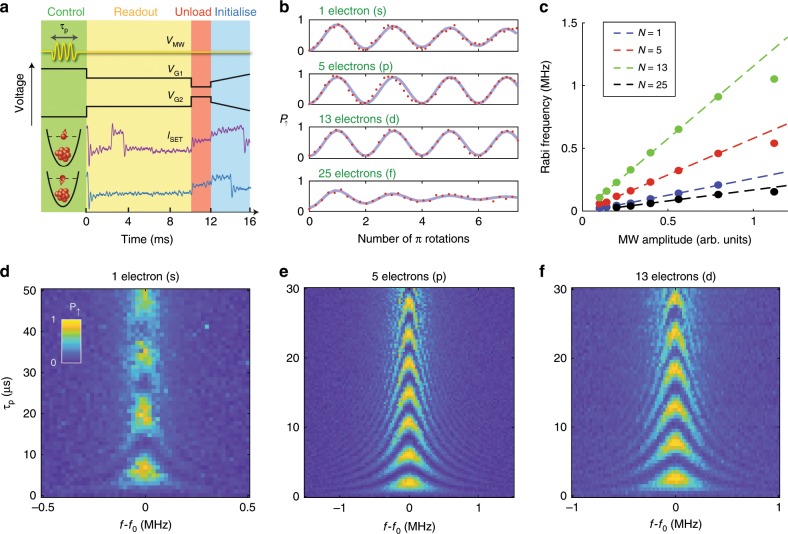
Coherent spin control. **a** Gate and microwave pulse sequence for single-qubit control and read-out. The lower section shows the change in SET current when a valence electron is in either a spin up or down state. **b** Rabi oscillation of the probability of measuring a spin up *P*_*↑*_ for *N* = 1, 5, 13 and 25 electrons, under the same driving power from the microwave source. Traces for s, p, d electrons are extracted from (**d**–**f**) at *f*  = *f*_0_, fitted using ${P}_{\uparrow }=A\, \, {\mathrm{cos}}(2\pi {f}_{{\rm{Rabi}}}t){e}^{-t/{T}_{2}^{{\rm{Rabi}}}}+c$, where *A* and *c* are related to the measurement visibility. Horizontal axis is number of *π* rotations ($\frac{{\tau }_{p}}{{T}_{\pi }}$) of each oscillation. **c** Rabi frequencies as a function of applied microwave power, at different electron numbers *N*. **d**–**f** Probability of spin up as a function of ESR frequency detuning and duration of microwave pulse for (**d**) *N*  =  1, (**e**) *N*  = 5 and (**f**) *N* = 13 electrons, performed along the grey dashed line in Fig. 3e, f, h, i, k, l, which correspond to the highest Q-factor operating points for each electron occupancy. Resonance frequencies *f*_0_ for *N*  =  1, 5 and 13 are 41.829, 41.879 and 41.827 GHz, respectively.

In order to initialize, control and read-out the spins, the pulse sequence depicted in Fig. 2a is performed. The amplitude and duration of the driving AC electric field is used to implement various single-qubit logical gates. The fidelity of these qubit operations under the decoherence introduced by the environment is probed by a randomized benchmarking protocol^36,37^, shown in the Supplementary Fig. 3 for s-, p-, and d-electrons. Single-qubit elementary gate fidelities improve from 98.5% to 99.7% and 99.5% when the electron occupancy increases from 1 to 5 and 13 electrons. Part of the reason for this improvement is the reduction of the quantum dot confinement at higher occupations—the Coulomb repulsion due to electrons in inner shells leads to a shallower confinement, thus reducing charging and orbital energies (Fig. 1d) and ultimately leading to faster Rabi frequencies *f*_Rabi_. We note that this effect cannot be compensated by an increase in the driving power (amplitude of the oscillating electric field) because the Rabi frequency saturates at high power (Fig. 2c).

At s-, p-, d-, and f-electron occupations, Rabi frequencies increase linearly with microwave amplitude (Fig. 2c). As more electrons occupy the quantum dot, their wavefunction size increases and confinement energy decreases, hence leading to a higher effective oscillating magnetic field via EDSR^33^. At the f-electron, other effects such as multiple relaxation hot spots prevent an optimal voltage configuration for the qubit, resulting in a inefficient Rabi drive.

One should note that the Dresselhaus spin-orbit coupling has very distinct impact on each valley state, which could potentially affect EDSR if it was driven by the material spin-orbit coupling^38,39^. Since our EDSR approach adopts an inhomogeneous magnetic field induced by a micromagnet, however, we expect that possible suppressions of spin-orbit effects by valley interference are overcome by the field gradient. In other words, the observed improvement of the Rabi oscillations is unlikely to largely stem from variations in the valley structure among shells.

A more intuitive way to probe the effects of faster gating times is by measuring the *Q*-factor ($Q={T}_{2}^{{\rm{Rabi}}}/{T}_{\pi }$) of Rabi oscillations of 1, 5, 13 and 25 electrons (see Fig. 2b). The amplitude for s and f electrons is damped quickly enough that after 7*π* rotations a noticeable decrease in coherence is observed. We extract *Q* = 14 for s electrons and *Q* = 3.4 for f electrons. Conversely, p and d electrons show barely visible decay. The minimum observed *Q*-factor for either p or d electrons, obtained in a different voltage configuration, was *Q* > 34 (this value is strongly impacted by gate bias voltages and power of the EDSR driving field). This effect also cannot be compensated with the driving power because the *Q*-factor is not significantly improved at any particular value of the microwave amplitude (see Supplementary Note 2).

Moreover, Rabi chevron plots in Fig. 2d–f show a visible improvement in the quality of both *N* = 5 and 13 electrons compared with *N* =  1. Further coherence time measurements were also performed, with $$ {T}_{2}^{* }$$ ranging from 5.7 to 18.1 μs and $$ {T}_{2}^{{\rm{Hahn}}}$$ between 21.6 and 68.5 μs (see Supplementary Note 4 for details)^40,41^. We highlight that the coherence times of p and d electrons still outperform the single spin coherence obtained in natural silicon^42^, indicating that closed shell electrons are not a leading source of dephasing noise. The direct quantitative comparison between coherence times is not a precise measure of robustness against noise because the total acquisition time may impact the estimate of $$ {T}_{2}^{* }$$, but we conclude that no impact on $$ {T}_{2}^{* }$$ is observed due to the electrons which comprise a closed shell. The small variations in coherence are largely compensated by the enhanced Rabi frequency for p and d electrons, which explains the improved qubit performances.

Although Rabi oscillations are visible for *N* = 25 in Fig. 2b, we observed its optimal *π*-pulse time and ${T}_{2}^{{\rm{Rabi}}}$ to be similar to *N* = 1. This indicates that higher shell numbers do not necessary benefit qubit operation, as more relaxation hot spots will arise with the increased multiplicity of the shell states^12^.

### Impact of excited states on multielectron qubits

**Fig. 3:**
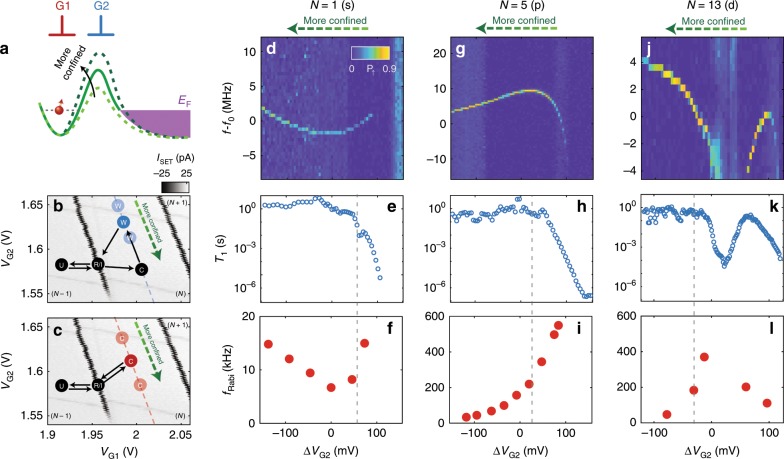
Stark shift, tunable Rabi frequency and relaxation time. **a** Schematic representation of the quantum device energy band diagram. G2 voltage varies in order to change the quantum dot size and tunnel rate to the reservoir (purple). Compensating voltage is also applied to G1 to maintain the quantum dot energy level relative to the Fermi level *E*_F_. **b**, **c** Schematics of the pulse sequences for (**b**) *T*_1_ relaxation, and (**c**) Rabi control experiment. The qubit control point varies along the dashed line inside the charge stability diagram, parallel to the charge transitions, with electron occupancy either *N* = 1, 5 or 13. **d** Non-linear Stark shift of qubit resonance frequency is observed when the qubit control point changes along dashed line in (**b**) and (**c**), for *N* = 1 electron. At certain voltage levels, the resonance frequency shifts dramatically and eventually qubit read-out is unachievable. **e** Correlation is observed between the magnitude of the differential ESR resonance frequency (*f*  − *f*_0_) and qubit relaxation time *T*_1_. **f** Correlation is also observed between *f* − *f*_0_ and Rabi frequency *f*_Rabi_. There is a qualitative correlation between the maximum Rabi frequency and the non-linearity of the Stark shift. **g**–**l** Stark shift, *T*_1_ and Rabi frequencies as plotted in Figs. (**d**–**f**), but for (**g**–**i**) *N* = 5 and (**j**–**l**) 13 electrons. Examples of advantageous qubit operation voltages, where a balance exists between fast Rabi oscillation and long spin lifetime, are drawn as grey vertical lines in the figure. *f*_0_ = 41.835, 41.870, 41.826 GHz for *N* = 1, 5 and 13 electrons, respectively.

Although multielectron quantum dots can be exploited to improve qubit performance, they raise new questions regarding the many-body physics of these dots. One particular concern is that the presence of low-lying excited orbital states may interfere with the spin dynamics. We track the excited states by altering the dot aspect ratio without changing its occupancy^43^ (see schematic in Fig. 3a), by adjusting the G1 and G2 gate voltages as indicated in Fig. 3b and c. We first measure the qubit resonance frequencies while varying the dot shape (Fig. 3c). This frequency is impacted by variations in g-factor and micromagnet field as the dot is distorted by the external electric field—we collectively refer to these effects as Stark shift. Linear Stark shift should be observed since the control point of the quantum dot is far detuned from any charge transition. Instead, non-linear Stark shifts are observed for *N* = 1 (Fig. 3d), *N* = 5 (Fig. 3g) and *N* = 13 electrons (Fig. 3j). Although such phenomenon can be partially explained by change in magnetic field experienced by the quantum dot along the *x*-direction, a significant drop in resonance frequencies is observed for *N* = 5 (Fig. 3g) and 13 electrons (Fig. 3j) at *ΔV*_G2_ > 100 mV and 20 mV < *ΔV*_G2_ < 60 mV, respectively.

To investigate this further, we measure the spin relaxation time *T*_1_ using the pulse sequence in Fig. 3b, as shown in Fig. 3e, h, k. A clear correlation between the drop in *T*_1_ and regions with a highly non-linear Stark shift is similar to previous literature^12,44,45^. This indicates the presence of an excited orbital or valley state nearby the Zeeman excitation, resulting in a reduction of *T*_1_.

Since the virtual excited state (either valley^46^ or orbital^47,48^) plays an essential role in EDSR, the excitation energy directly influences the qubit Rabi frequencies. Performing the pulse sequence in Fig. 3c, we observe an enhancement of one order of magnitude for the Rabi frequencies of p and d orbitals (Figs. 3f, i, l) correlated to the drop in *T*_1_. We also notice that the Larmor frequency and the Rabi frequency as a function of *ΔV*_G2_ in this experiment may be non-monotonic in some charge configurations, with a discernible correlation between their extrema. These are indications that the p and d spins are coupled to excited states of a different nature to those for s electrons. There are no charge transitions (or visible features in the charge stability diagram), indicating that the ground state configuration is left unchanged. Note that some Rabi frequency enhancement is also observed for the *N* = 1 electron configuration, but it is an order of magnitude lower than for *N* = 5 and 13 electrons.

We may exploit this control over the excitation spectrum to induce fast relaxation on demand for qubit initialization, to operate the qubit where *f*_Rabi_ is high, and to store it in a configuration where *T*_1_ is long. The power of the EDSR drive only impacts the observed *Q*-factor value up to a factor of 2 (see Supplementary Fig. 2), in contrast to recent observations in depletion mode quantum dot experiments^49^ where an order of magnitude difference in *Q*-factors were observed.

The additional relaxation hotspot around *ΔV*_G1_ = 10 mV for the d-shell qubit in Fig. 3k is most likely due to the increased number of near-degenerate orbitals present, which implies more pathways for qubit relaxation. This near-degeneracy could also be related to why the 14 electron configuration follows Hund’s rule to give a *S* = 1 ground state^9,50^ (see Fig. 1f). We note that these higher total spin states are observed to also be coherently drivable, but a detailed study of these high-spin states exceeds the scope of our present work (see Supplementary Fig. 5c & d).

## Discussion

The results presented here experimentally demonstrate that robust spin qubits can be implemented in multielectron quantum dots up to at least the third valence shell. Their utility indicates that it is not necessary to operate quantum dot qubits at single-electron occupancy, where disorder can degrade their reliability and performance. Furthermore, the larger size of multielectron wavefunctions combined with EDSR can enable higher control fidelities, and should also enhance exchange coupling between qubits^51^. A multielectron system results in a richer many-body excitation spectrum, which can lead to higher Rabi frequencies for fast qubit gates and enhanced relaxation rates for rapid qubit initialization. Future experiments exploring two-qubit gates using multielectron quantum dots will extend this understanding of electronic valence to interpret bonding between neighbouring dots in terms of their distinct orbital states. The controllability of the excitation spectrum should also allow for different regimes of electron pairing, including a possible singlet-triplet inversion^50^, mimicking the physics of paramagnetic bonding^52^.

## Supplementary information

Supplementary Information

## Data Availability

The data that support the findings of this study are available from the authors on reasonable request, see author contributions for specific data sets.

## References

[1] Vandersypen LMK (2017). Interfacing spin qubits in quantum dots and donors - hot, dense, and coherent. npj Quantum Inf..

[2] Hu X, Das Sarma S (2001). Spin-based quantum computation in multielectron quantum dots. Phys. Rev. A.

[3] Barnes E, Kestner JP, Nguyen NTT, Sarma SD (2011). Screening of charged impurities with multielectron singlet-triplet spin qubits in quantum dots. Phys. Rev. B.

[4] Bakker MA, Mehl S, Hiltunen T, Harju A, Di-Vincenzo DP (2015). Validity of the single-particle description and charge noise resilience for multielectron quantum dots. Phys. Rev. B.

[5] Harvey-Collard P (2017). Coherent coupling between a quantum dot and a donor in silicon. Nat. Commun..

[6] Tarucha S, Austing DG, Honda T, Van Der Hage RJ, Kouwenhoven LP (1996). Shell filling and spin effects in a few electron quantum dot. Phys. Rev. Lett..

[7] Kouwenhoven LP (1997). Excitation spectra of circular, few-electron quantum dots. Science.

[8] Rontani M (2006). Full configuration interaction approach to the few-electron problem in artificial atoms. J. Chem. Phys..

[9] Deng K, Calderon-Vargas F, Mayhall NJ, Barnes E (2018). Negative exchange interactions in coupled few-electron quantum dots. Phys. Rev. B.

[10] Malinowski FK (2018). Spin of a multielectron quantum dot and its interaction with a neighboring electron. Phys. Rev. X.

[11] Yang CH, Lim WH, Zwanenburg FA, Dzurak AS (2011). Dynamically controlled charge sensing of a few-electron silicon quantum dot. AIP Adv..

[12] Yang CH (2013). Spin-valley lifetimes in a silicon quantum dot with tunable valley splitting. Nat. Commun..

[13] Fock V (1928). Bemerkung zur Quantelung des harmonischen Oszillators im Magnetfeld. Z. für. Phys. A Hadrons Nucl..

[14] Darwin, C. G. in *Mathematical Proceedings of the Cambridge Philosophical Society*, vol. 27, 86–90 (Cambridge University Press, 1931).

[15] Zwanenburg FA (2013). Silicon quantum electronics. Rev. Mod. Phys..

[16] Ngo CY, Yoon SF, Fan WJ, Chua SJ (2006). Effects of size and shape on electronic states of quantum dots. Phys. Rev. B.

[17] Harting J, Mülken O, Borrmann P (2000). Interplay between shell effects and electron correlations in quantum dots. Phys. Rev. B.

[18] Lim WH (2011). Spin filling of valley-orbit states in a silicon quantum dot. Nanotechnology.

[19] Borselli MG (2011). Measurement of valley splitting in high-symmetry Si/SiGe quantum dots. Appl. Phys. Lett..

[20] Yang CH (2012). Orbital and valley state spectra of a few-electron silicon quantum dot. Phys. Rev. B.

[21] Jacak, L., Hawrylak, P., Wojs, A., Wójs, A. & Wojs, A. *Quantum Dots* (Springer Science & Business Media, 2013).

[22] Folk, J. et al. in *Quantum Chaos Y2K*, 26–33 (World Scientific, 2001).

[23] Haldane FDM (1983). Continuum dynamics of the 1-d heisenberg antiferromagnet: identification with the o (3) nonlinear sigma model. Phys. Lett. A.

[24] Brennen GK, Miyake A (2008). Measurement-based quantum computer in the gapped ground state of a two-body hamiltonian. Phys. Rev. Lett..

[25] Bartlett SD, Brennen GK, Miyake A, Renes JM (2010). Quantum computational renormalization in the haldane phase. Phys. Rev. Lett..

[26] Miyake A (2010). Quantum computation on the edge of a symmetry-protected topological order. Phys. Rev. Lett..

[27] Senko C (2015). Realization of a quantum integer-spin chain with controllable interactions. Phys. Rev. X.

[28] Higginbotham AP, Kuemmeth F, Hanson MP, Gossard AC, Marcus CM (2014). Coherent operations and screening in multielectron spin qubits. Phys. Rev. Lett..

[29] Elzerman JM (2004). Single-shot read-out of an individual electron spin in a quantum dot. Nature.

[30] Koppens FHL (2006). Driven coherent oscillations of a single electron spin in a quantum dot. Nature.

[31] Veldhorst M (2014). An addressable quantum dot qubit with fault-tolerant control-fidelity. Nat. Nanotechnol..

[32] Pla JJ (2012). A single-atom electron spin qubit in silicon. Nature.

[33] Pioro-Ladrière M (2008). Electrically driven single-electron spin resonance in a slanting Zeeman field. Nat. Phys..

[34] Tokura Y, Van Der Wiel WG, Obata T, Tarucha S (2006). Coherent single electron spin control in a slanting zeeman field. Phys. Rev. Lett..

[35] Kawakami E (2014). Electrical control of a long-lived spin qubit in a Si/SiGe quantum dot. Nat. Nanotechnol..

[36] Knill E (2008). Randomized benchmarking of quantum gates. Phys. Rev. A.

[37] Magesan E, Gambetta JM, Emerson J (2011). Scalable and robust randomized benchmarking of quantum processes. Phys. Rev. Lett..

[38] Nowack KC, Koppens FHL, Nazarov YV, Vandersypen LMK (2007). Coherent control of a single electron spin with electric fields. Science.

[39] Corna A (2018). Electrically driven electron spin resonance mediated by spin-valley-orbit coupling in a silicon quantum dot. npj Quantum Inf..

[40] Kha A, Joynt R, Culcer D (2015). Do micromagnets expose spin qubits to charge and Johnson noise?. Appl. Phys. Lett..

[41] Zhao, R. et al. Single-spin qubits in isotopically enriched silicon at low magnetic field. *Nat. Commun*. **10**, 5500 (2019). 10.1038/s41467-019-13416-7 PMC6890755 31796728

[42] Watson T (2018). A programmable two-qubit quantum processor in silicon. Nature.

[43] Hwang JCC (2017). Impact of g-factors and valleys on spin qubits in a silicon double quantum dot. Phys. Rev. B.

[44] Srinivasa V, Nowack KC, Shafiei M, Vandersypen LMK, Taylor JM (2013). Simultaneous spin-charge relaxation in double quantum Dots. Phys. Rev. Lett..

[45] Borjans F, Zajac DM, Hazard TM, Petta JR (2019). Single-spin relaxation in a synthetic spin-orbit field. Phys. Rev. Appl..

[46] Hao X, Ruskov R, Xiao M, Tahan C, Jiang H (2014). Electron spin resonance and spin-valley physics in a silicon double quantum dot. Nat. Commun..

[47] Amasha S (2008). Electrical control of spin relaxation in a quantum dot. Phys. Rev. Lett..

[48] Rashba EI (2008). Theory of electric dipole spin resonance in quantum dots: mean field theory with Gaussian fluctuations and beyond. Phys. Rev. B.

[49] Takeda K (2016). A fault-tolerant addressable spin qubit in a natural silicon quantum dot. Sci. Adv..

[50] Martins F (2017). Negative spin exchange in a multielectron quantum dot. Phys. Rev. Lett..

[51] Yang, C. et al. Silicon quantum processor unit cell operation above one kelvin. Preprint at https://arxiv.org/abs/1902.09126 (2019). 10.1038/s41586-020-2171-6 32296190

[52] Lange KK, Tellgren EI, Hoffmann MR, Helgaker T (2012). A paramagnetic bonding mechanism for diatomics in strong magnetic fields. Science.

