# Supplementary material for: Coherent spin control of s-, p-, d- and f-electrons in a silicon quantum dot
Source: Nat Commun. 2020 Feb 11;11:797. doi: 10.1038/s41467-019-14053-w (PMC7012832; doi:10.1038/s41467-019-14053-w)
Supplement: Supplementary file 1 — Supplementary Information [file 41467_2019_14053_MOESM1_ESM.pdf]

Supplementary Material: Coherent spin control of s-, p-, d- and f-electrons in a silicon quantum dot

Leon et al.

Contents

Supplementary Note 1: Micromagnet design 2

Supplementary Note 2: Rabi frequencies and power dependence of Q-factor 2

Supplementary Note 3: Gate voltage tuning of the Q-factor 2

Supplementary Note 4: Coherence of various quantum dot fillings 3

Supplementary Note 5: Coherent spin control at other electron occupations 4

References 4

Supplementary Note 1: Micromagnet design

A layer of 460 μm long, 600 nm wide, 250 nm thick cobalt is deposited next to the Si-MOS structure shown in Fig. 1 of the main text. The cobalt width increases at regions further away from quantum dot, up to 80 μm, resulting in a trapezoidal prism structure. A 10 nm thick titanium layer is deposited before cobalt to ensure adhesion between SiO_2 and cobalt during deposition.

All experiments presented in the main text of the paper are carried at B_0 =1.4 T. Magnetic field simulations performed with Radia package of Mathematica (Supp. Fig. 1) indicate that the magnet produces a gradient of ~ 1 T/μm at the quantum dot location. This value can be considered constant in all experiments. Indeed, the quantization axis is well defined by the external field and the magnet is fully magnetized at such large field.

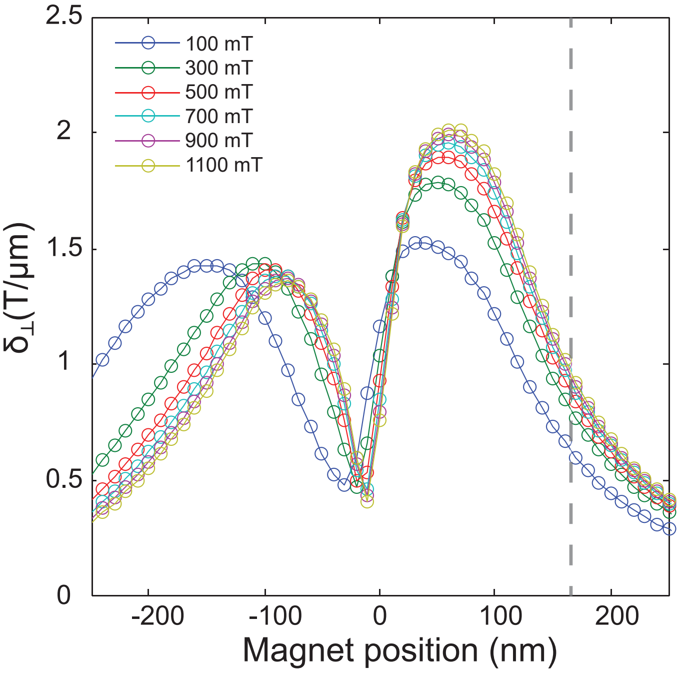

Supplementary Figure 1: **Magnetic field gradient produced by micromagnet.** Simulation of the transverse magnetic field gradient produced by the cobalt layer[1], at various applied external magnetic fields B_0. The grey dashed line indicates the position of the quantum dot.

Supplementary Note 2: Rabi frequencies and power dependence of Q-factor

Although variations of the Q-factor ($Q = T_2^{\mathrm{Rabi}}/T_\pi$) are observed for different electron occupancy, the MW amplitude dependence remains unclear. Previous work in Ref.2 has identified operation points where the applied microwave power maximizes the Q-factor. From values and error bars in Supp Fig. 2, it is not possible to identify such optimal operation point.

Supplementary Note 3: Gate voltage tuning of the Q-factor

As mentioned in the main text and Supp. Fig. 4a, qubit Rabi frequencies depend on the degree of confinement of the quantum dot, controlled by the gate voltages. Changing quantum dot confinement, on the other hand, may also increase its exposure to various noises. In Supp. Fig. 4b, T_2^{Rabi} varies up to an order of magnitude with changes in confinement level of the p-electron. The observed T_2^{Rabi} appears to be inversely proportional to $|\Delta f/\Delta V_{G2}|$ in Fig. 3g. This suggests that electric noise is an important source of dephasing, coupling to the spin qubit through the Stark shift. As a result of Supp. Fig. 4a and b, an optimal operation point is extracted with a maximum quality factor Q, as shown in Supp. Fig. 4c. Note that the d-electron qubit shares a similar behaviour as the p-electron, while the s-electron does not have any observable change in Q due to the lack of excitation hotspots.

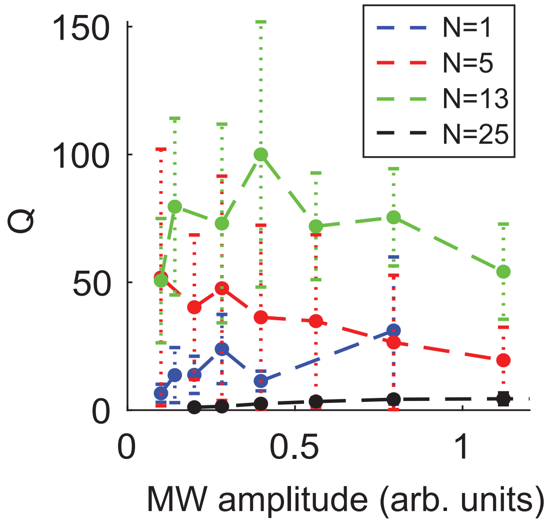

Supplementary Figure 2: **Microwave power dependence.** Q-factor as a function of applied microwave amplitude, at 1, 5, 13, 25 electrons configuration. Error bar represents ±5% error.

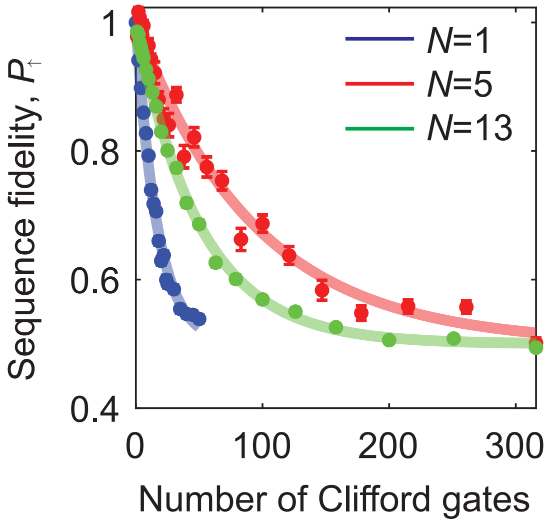

Supplementary Figure 3: **Randomised benchmarking.** Single qubit randomised benchmarking for $N = 1$, 5 and 13 electrons, showing the sequence fidelity $P_\uparrow$ as a function of the number of applied Clifford gates, providing elementary gate fidelities of 98.5 %, 99.7 % and 99.5 %, respectively. Error bars represent the standard error of the mean.

Supplementary Note 4: Coherence of various quantum dot fillings

The coherence time T_2^* is not significantly affected at different electron occupancies as seen in Table I. The main source of decoherence is electrical noise under magnetic field gradients[3]. Assuming a quantum dot radius of $a = 20$ nm, using equation (5) in Ref. 3, with in plane magnetic field gradient $\delta_\parallel = 0.8$ T/μm, we estimated $T_2^* \sim 50$ μs. Study in Ref. 4 reveals that decoherence due to 800ppm residual silicon-29 nuclei in an isotopically purified silicon wafer sets T_2^* to 10-100 μs, meaning it can possibly be a contributing factor of decoherence.

Again, in Table I, T_2^{Hahn} of all electron occupancies are within an order of magnitude, with T_2^{Hahn} of N =1 larger than $N = 5$ and 13 by a factor of 3. The larger coupling to charge noise could result in this lesser effect of the Hahn echo sequence. Interestingly, refocusing using Hahn echo is more beneficial to $N = 13$ than 5, possibly due to the differences between p-orbitals and d-orbitals. This is supported by a higher quality factor in $N = 13$ of Fig. 2b, where high frequency noises are filtered. On the other hand, the lower overall noise level in T_2^* data at $N = 5$ than 13 agrees with the higher fidelity of the Clifford gates.

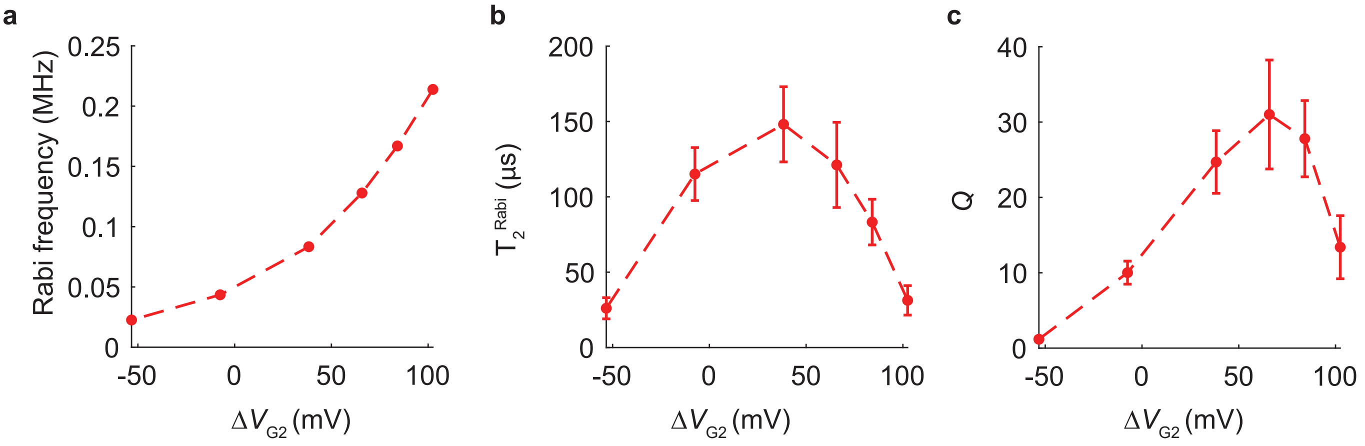

Supplementary Figure 4: **Confinement voltage dependent Q-factor.** **(a)** Rabi frequency, **(b)** T_2^{Rabi} and **(c)** Q-factor as a function of change in gate voltage ΔV_{G1} and ΔV_{G2}, following the dashed line in Fig. 3c of main text, at 5 electrons configuration. Error bar represents ±5% error.

Number of electrons	$T_2{}^*$ (μs)	T_2^{Hahn} (μs)
1	18	69
5	16	22
13	7.8	29

TABLE I: Decoherence time of qubits at various electron occupancies.

Supplementary Note 5: Coherent spin control at other electron occupations

Besides fillings consisting of single valence electron, we also coherently control the qubit at other electron occupations - namely 3, 9, 10, 14 and 27 electrons, as shown in Supp. Fig. 5.

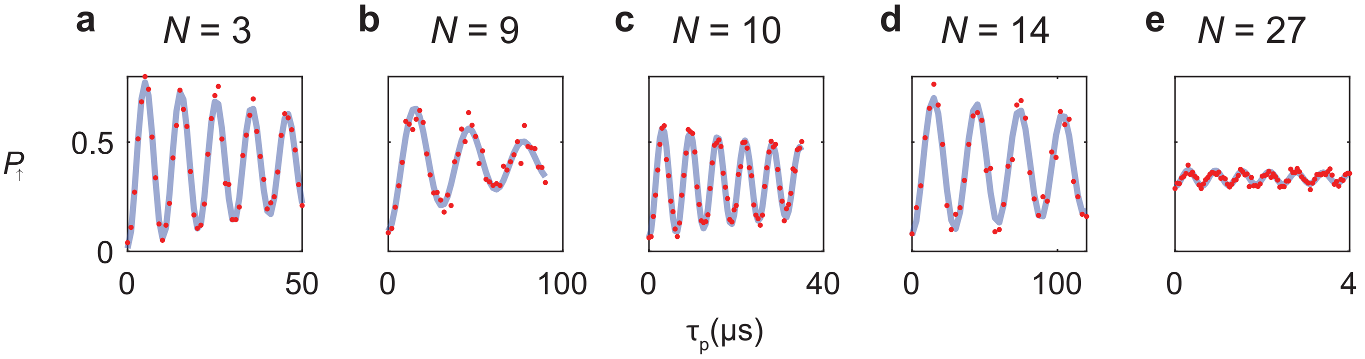

Supplementary Figure 5: **Coherent control at various electron occupancies.** Rabi oscillations in the spin up probability $P_\uparrow$ for different electron numbers N inside the a single quantum dot. **(a)** $N = 3$ **(b)** $N = 9$ **(c)** $N = 10$ **(d)** $N = 14$ **(e)** $N = 27$. Note that from Fig. **??**f, N =10 and 14 electrons have total spin states $S = 1$, while N =27 electrons has $S = \frac{3}{2}$.

[1] Wolfram Research, I. Mathematica (2018).

[2] Takeda, K. *et al.* A fault-tolerant addressable spin qubit in a natural silicon quantum dot. *Science Advances* **2**, e1600694 (2016).

[3] Kha, A., Joynt, R. & Culcer, D. Do micromagnets expose spin qubits to charge and Johnson noise? *Applied Physics Letters* **107**, 172101 (2015).

[4] Zhao, R. *et al.* Coherent single-spin control with high-fidelity singlet-triplet readout in silicon. *Preprint at https://arxiv.org/abs/1812.08347* (2019).
